# Rosai-Dorfman Disease: A previously unreported association with Sickle Cell Disease

**DOI:** 10.1186/1472-6890-7-3

**Published:** 2007-04-02

**Authors:** Claire Stebbing, Jon van der Walt, Ghada Ramadan, Baba Inusa

**Affiliations:** 1 Specialist Registrar in Pediatrics, Guy's and St Thomas' Hospital, Lambeth Palace Road, London, SE1 7EH, UK; 2 Consultant Histopathologist, Guy's and St Thomas' Hospital, Lambeth Palace Road, London, SE1 7EH, UK; 3 Consultant Pediatrician with special interest in hemoglobinopathies, Guy's and St Thomas' Hospital, Lambeth Palace Road, London, SE1 7EH, UK

## Abstract

**Background:**

Rosai-Dorfman Disease is an uncommon benign systemic histio-proliferative disease. This is the first time the disease, although more common in people of African descent, is described in association with Sickle cell disease.

**Case presentation:**

A Nigerian boy born started a complex medical history with post-natal anemia of unknown origin. Subsequently he was diagnosed with Sickle Cell Anemia (Hb SS). At age 3 during a routine review, he was noted to have generalised massive lymphadenopathy. He had further reoccurrences of this lymphadenopathy, but investigations did not reveal the cause until age five. At this point, because of the progressive lymph node enlargement, a biopsy was performed, and he was diagnosed with Rosai-Dorfman Disease. Since that time, the child has had further episodes of intermittent massive lymphadenopathy, particularly associated with Sickle Crisis. His medical history has been further complicated by development of complications from Sickle Cell Disease, cardiomyopathy and an autoimmune hemolytic anemia with multiple alloantibodies.

**Conclusion:**

This case for the first time presents the co-existence of two diseases, of increased prevalence in those of African descent, but to date not described in the literature to occur concurrently.

## Background

Sinus Histiocytosis with Massive Lymphadenopathy otherwise known as Rosai-Dorfman Disease, (RDD), is an uncommon, benign systemic histio-proliferative disease characterised by massive lymphadenopathy, particularly in the head and neck region, and often associated with extra nodal involvement [1]. Sickle cell disease (SCD) is a common disorder predominantly in people of African origin. Although RDD is more common in Africans [1], this is the first case presentation of a child with SCD and RDD. The child first presented with severe anemia of unknown cause at the age of six weeks and has since had recurrent infections. He has now developed features of autoimmune hemolytic anemia.

## Case presentation

A three-year-old boy, of Nigerian parents, with Sickle Cell Anemia (Hb SS), with HbF 8–14%, and steady state Hb 6–7 g/dl, diagnosed at birth. The diagnosis of SCD was confirmed at 3 months of age, with Hb electrophoresis showing bands predominantly at S, F and absent A. He was admitted to the hospital at the age of 6 weeks, with severe anemia of unknown cause for which he received the first blood transfusion. He then presented for routine follow up at the age of three years and found to have massive lymph node enlargement. Nodes were noted in the deep cervical, posterior auricular and axillary chains. The largest measured 2 cm by 2 cm. Nodes were also present in both groins, measuring about 1.5 cm, and were mobile and soft. There was no evidence of ongoing systemic illness. Examination was otherwise unremarkable, and there was no hepatosplenomegaly. An ultrasound at this time also confirmed the presence of lymph nodes at the hilum of the spleen and liver. No diagnosis was made and the nodes appeared to reduce in size and were presumed to be the result of a viral infection or sickle cell disease.

Later in the same year, he was admitted with acute vaso-occlusive crisis. At this time, the lymph node enlargement was again noted. Further radiological investigation confirmed the multiple enlarged lymph nodes scattered throughout the abdomen. Other investigations, including a complete blood count, blood film, chest radiograph, virology screen, lymphocyte sub-sets, Heaf test (Screening for Tuberculosis) and HIV test, were all normal. The lymphadenopathy once again started to resolve spontaneously.

**Figure 1:**
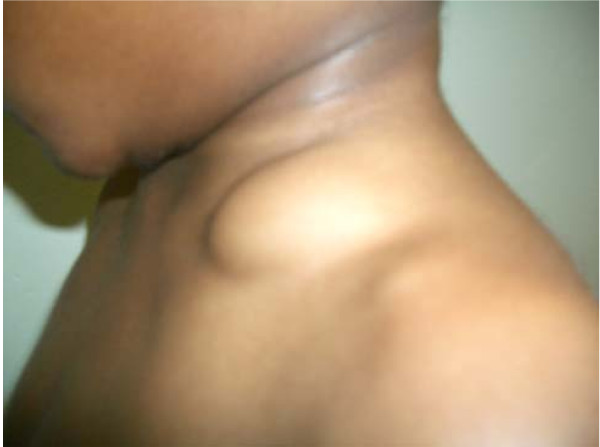
Photographs of the child at age 5, showing massive lymphadenopathy.

At age five, he developed further recurrence of the lymphadenopathy. Examination, at this time revealed multiple enlarged lymph nodes in axillary, cervical and inguinal groups. The lymph nodes were attached to each other, with a 'grape-like' appearance and 'sponge-like' consistency on palpation. The lymph node enlargement is shown in Figure 1. Because of the progressive lymph node enlargement, an open lymph node biopsy under anaesthesia was performed, which was reported as follows:

There was hyperplasia of the paracortex with abundance of plasma cells. In addition, there were very large epitheliod histiocytes, which contained lymphoid cells within their cytoplasm. Immunohistochemistry showed this plasma cell population to be polyclonal. The histiocytes stained with S100 protein typical of the diagnosis of RDD.

**Figure 2:**
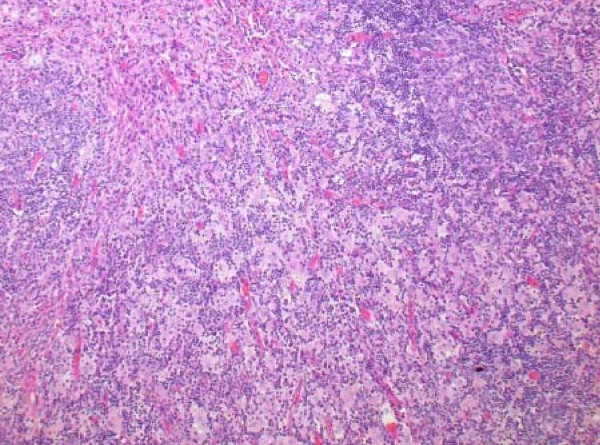
Lower power showing large epithelioid histiocytes scattered throughout the Paracortex.

**Figure 3:**
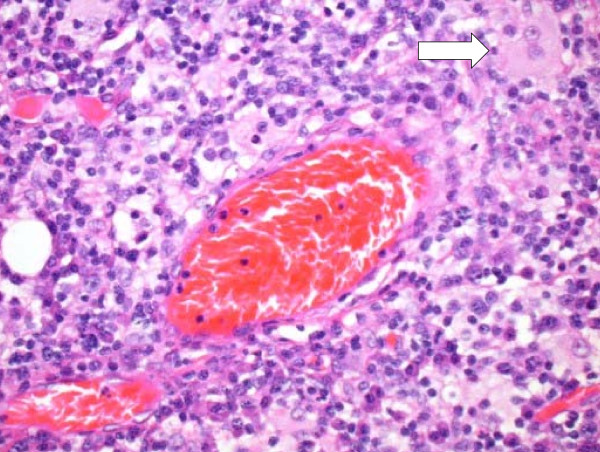
High Power showing sickled red cells in a vessel and details of the epithelioid histiocytes (white arrow) which contain lymphocytes within their cytoplasm.

The histological appearances are shown in Figures 2, 3.

Subsequently, his medical history has been complicated by repeated admissions for infections and vaso-occlusive crises and the need for chronic blood transfusion for three years. This in turn led to iron overload due to non-compliance with chelation therapy and so the blood transfusions were stopped. In addition shortly after the second presentation with massive lymphadenopathy, he developed overwhelming Streptococcus pneumoniae septicemia, requiring ventilation. This was further complicated by cardiac failure, secondary to dilated cardiomyopathy. He is no longer on blood transfusions, but continues on cardiac medication.

At the age of seven, he again developed severe anemia with hemoglobin of 2.6 gm/dl, associated with Morganella morganii septic arthritis, from which he recovered after prolonged antibiotic treatment. The infectious complications are most probably related to the SCD. He also recently developed a hemolytic anemia with a positive Direct Coombs Test (DCT) and a reticulocyte count of 330 × 10^9^/l. The auto-antibodies are not thought to be linked to the transfusion programme because whilst on the transfusion programme he had repeated negative DCT's, until 8 months after cessation of the transfusion programme. The degree of DCT has progressed from mildly to strongly positive, and there are now demonstrable anti-bodies e.g. IgG5 and IgGB.

## Conclusion

RDD usually presents with painless massive cervical lymphadenopathy, rarely the first presentation is with extra nodal manifestations [1]. The disease appears to be reactive because the histocytic proliferation is polyclonal [2]. Histiocytic proliferation is thought to be secondary to abnormal cytokine production, leading to the typical Rosai-Dorfman cells [2]. The diagnostic appearances are due to hyperplasia of the lymph nodes with polymorphous cell proliferation of lymphoid cells and characteristic histiocytes (Rosai-Dorfman cells) [3]. The histiocytes may contain intact lymphocytes or rarely other cells within the cytoplasm – empeiripolesis [1].

The histiocytes are positive for S-100 protein and appear to be activated [3]. The architecture of the nodes is not disrupted until very late in the disease cycle.

The disease is most common in adolescents and young adults, and is particularly found amongst the African and Caribbean population [1]. The lymphadenopathy is most commonly seen in the head and neck but in rare cases, the disease presentation is extranodal [1]. The disease is self-limiting with 70–80% undergoing spontaneous remission while other cases may be chronic with a relapsing and remitting pattern [3]. Surgery is only required when the lymph node enlargement erodes into surrounding vital organs such as heart, lungs and endocrine organs. Other therapeutic options may be considered in these situations. There have been trials of chemotherapy and or radiation therapies with variable outcome, not very successful in the majority of cases [3]. There are recent reports of 2-CdA, a purine analogue more commonly used for Langerhans Cell Histiocytosis (LCH), trialed for use in recurrent RDD, providing only symptomatic relief [2].

RDD is an uncommon disease, not previously reported in a child with SCD. It may be difficult to diagnose particularly if, as in this case, the disease waxes and wanes with long periods of disease remission lasting years. SCD is also known to be associated with generalised lymph node enlargement, but is unlikely to explain massive gland enlargement. RDD is much easier to diagnose when it is associated with massive enlargement of cervical nodes and a high index of suspicion. This patient has shown some unusual features: early presentation and cardiomyopathy possibly due to extra-nodal disease. Equally, his initial presentations at the age of three and three and a half, whilst retrospectively suspicious of RDD, cannot conclusively be proven to be so, as no tissue diagnosis exists from then. His case is further complicated by the undiagnosed severe anemia at the age of six weeks, which, was unlikely to be the result of SCD, in the presence of high fetal hemoglobin; recurrent opportunistic infection such as Morganella septic arthritis and the recent hemolytic anemia. However, there is no evidence of associated immunodeficiency [4].

We do not postulate a causative link between these two diseases rather a coincidental co-occurrence. However, the presence of both diseases and his other medical problems may lead to management challenges, although this has not proven to be the case to date. His management remains supportive with no treatment for the RDD. However, during his most recent episode of massive lymphadenopathy the use of steroids was considered because the lymphadenopathy was feared to be compromising respiration. His current pattern seems to that of recurrent massive lymphadenopathy often associated with sickle cell crisis.

## Competing interests

The author(s) declare that they have no competing interests.

## Authors' contributions

CS and BI conceived the idea for the case study. CS and GR performed the literature review. CS, BI and GR wrote the case presentation. CS, BI and JDW developed the design and coordinated the writing of the article. All read and approved the final manuscript.

## Pre-publication history

The pre-publication history for this paper can be accessed here:



## References

[B1] Foucar E, Rosai J, Dorfman R (1990). Sinus histiocytosis with massive lymphadenopathy (Rosai-Dorfman disease): review of the entity. Semin Diagn Pathol.

[B2] Rodriguez-Galindo C, Helton KJ, Sanchez ND, Rieman M, Jeng M, Wang W (2004). Extranodal Rosai-Dorfman disease in children. J Pediatr Hematol Oncol.

[B3] Carbone A, Passannante A, Gloghini A, Devaney KO, Rinaldo A, Ferlito A (1999). Review of sinus histiocytosis with massive lymphadenopathy (Rosai-Dorfman disease) of head and neck. Ann Otol Rhinol Laryngol.

[B4] Grabczynska SA, Toh CT, Francis N, Costello C, Bunker CB (2001). Rosai-Dorfman disease complicated by autoimmune haemolytic anaemia: case report and review of a multisystem disease with cutaneous infiltrates. Br J Dermatol.

